# Sensitization to rodents (mouse/rat) in urban atopic populations without occupational exposure living in Campania district (Southern Italy): a multicenter study

**DOI:** 10.1186/2049-6958-8-30

**Published:** 2013-04-16

**Authors:** Gennaro Liccardi, Gennaro Baldi, Anna Ciccarelli, Marina Cutajar, Maria D’Amato, Domenico Gargano, Domenico Giannattasio, Gennaro Leone, Mario Lo Schiavo, Francesco Madonna, Giovanni Menna, Carmen Montera, Antonio Pio, Maria Russo, Antonello Salzillo, Anna Stanziola, Gennaro D’Amato

**Affiliations:** 1 Department of Chest Diseases, Division of Pneumology and Allergology. High Speciality “A.Cardarelli” Hospital, Naples, Italy; 2 Respiratory Medicine Unit, ASL (District 66), Salerno, Italy; 3 Allergy Unit, Presidio Sanitario Polispecialistico “Loreto Crispi”, Naples, Italy; 4 Allergy Center, Division of Internal Medicine.,Ospedali Riuniti Penisola Sorrentina, Sorrento, Naples, Italy; 5 Department of Respiratory Disease, “Federico II” University – AO “Dei Colli”, Naples, Italy; 6 Allergy Unit. High Speciality “San Giuseppe Moscati” Hospital, Avellino, Italy; 7 Respiratory physiopathology and allergy,HighSpecialityCenter. “S.Maria Incoronata dell’Olmo” Hospital, Cava dei tirreni, Salerno, Italy; 8 Allergy and Clinical Immunology Unit. High Speciality “Sant’Anna and San Sebastiano” Hospital, Caserta, Italy; 9 Allergy and Clinical Immunology, “G. Fucito” Hospital and University Hospital, Salerno, Italy; 10 Allergy Unit, ASL (Sanitary District n°12), Caserta, Italy; 11 Animal Consultant, Naples, Italy; 12 Italian Association of Hospital and Territorial Allergologists (AAITO), Campania District, Southern Italy, Italy

**Keywords:** Airway hyperreactivity, Allergic rhinitis, Bronchial asthma, Campania district, Mouse, Rat, Pet Allergy

## Abstract

**Background:**

Up to now very few data on allergic sensitization to rodent allergens in Western Europe and Italy are available, and there are no information at district level.

The aim of this report was to investigate clinical significance and characteristics of allergic sensitization to mouse/rat (M/Rt) allergens in atopic subjects living in Campania district (Southern Italy).

**Methods:**

Allergists from the whole Campania district were required to report the results of skin prick tests of at least 100 consecutive subjects. In 1,477 consecutive outpatients, we selected all subjects with an immediate skin reaction to M/Rt dander. Clinical history including a careful evaluation of the modality of exposure and the results of skin-prick tests (SPTs) were recorded.

**Results:**

Fifty seven patients were sensitized to M/Rt dander (5.78%). Two patients were mono-sensitized. Fourteen patients reported indoor conditions suggesting presence of rodents allergens at home. All patients exhibited low-moderate degree of SPT positivity to M/Rt. High frequency of concomitant allergic sensitization to dust mites was found.

**Conclusions:**

Our results suggest that the role of allergic sensitization to rodents is not negligible in atopic subjects without occupational exposure living in Campania district area; these values are higher in comparison to those previously found in Naples area. Highly atopic individuals should be tested by SPTs/evaluation of serum specific IgE to rodents in the case they should begin an occupational exposure to M/Rt or keep these animals as pets.

## Background

Rodents (mouse and rats - M and Rt) are a well recognized cause of IgE-mediated sensitization and bronchial asthma in several occupationally exposed individuals such as research scientists, technicians and animal handlers [1]. More recently, it has been shown that M and Rt allergens play a significant role as airways sensitizing agents in atopic subjects also in indoor environments especially in some geographic areas such as United States [2-7]. On the contrary, very few studies on clinical aspects of rodent allergy have been published in other parts of the world [8-10]. Recently, we published the only study on rodent allergy in Italy and found that the prevalence of allergic sensitization to these animals is relatively low (1.60% for M and 0.59% for Rt) in Naples area [11]. However, since this value doesn’t necessarily reflect the true value of a larger territory such as the district area in which Naples is the chief town, we sought to perform a prospective study for assessing the prevalence of allergic sensitization, clinical characteristics and modality of exposure to common rodents (M/Rt) in a sample of atopic population without occupational exposure living in Campania district area (Southern Italy).

## Methods

Ten Allergy Units or Allergological Centres, uniformly distributed over the whole territory of Campania district (13,595 Km^2^, 6,074,882 inhabitants) participated in this cross-sectional study. Each centre was required to collect from January 1 to June 30, 2011 the results of at least 100 allergy consultations in consecutive outpatients referred for actual or suspected respiratory allergy (asthma and/or rhinitis).

1,477 subjects aged between 3 and 79 years (mean age 31.2) were examined.

All centres followed the same protocol**: **a case report form (CRF) containing all information, and specifically designed for this study, was completed during the screening consultation of each patient. The standardized form reported: demographic data, type and duration of respiratory symptoms, pets ownership, possible exposure to rodent allergens as assessed by some predictors (such as evidence of M/Rt/cockroach presence, poor housing conditions etc.), results of the skin prick tests (SPTs) for M/Rt dander. The forms were filled by the allergist, who also verified the consistency of clinical history and SPT results and the same doctor confirmed the diagnosis of respiratory allergy according to the International Guidelines [12,13]. Subjects with occupational exposure to rodents (workers exposed to laboratory animals in the pharmaceutical industry, university laboratories, research units, rodent breeding facilities or veterinary doctors) were not considered. We excluded also individuals working at mouse facilities including those non – mouse handling [14]. In order to avoid the passive transport of rodent allergens at home, patients living together relatives occupationally exposed to M/Rt were excluded too [15]. Patients with chronic infectious diseases, malignancies or dysmetabolic diseases, severe cutaneous disorders, negative skin reaction to histamine, or in treatment with drugs interfering with the skin response were excluded as well [16,17].

Since the absence of a pet at home does not exclude a direct exposure to pet outside [18] and considering the peculiarity of possible contacts with rodents, we classified animal exposure into two categories:

Positive contact: about pets, the presence of these animals at home or frequent direct contacts for different reasons (e.g. hobby, sport etc.), as regard rodents, predictors for presence of allergens in indoor environments such as evidence of M/Rt/cockroach presence, poor housing conditions [19] etc.

Negative contact: regarding pets, any direct pet contact but an indirect exposure though the contact with pet owners/any apparent direct or indirect exposure. Regarding rodents, any apparent predictors for presence of allergens in indoor environments.

The commercial allergen extracts used for screening SPTs were provided by Lofarma Laboratories, Milan Italy. We used a standard panel of allergens including: *Dermatophagoides pteronyssinus* and *D. farinae, Alternaria alternata, Cladosporium herbarum,* cat, dog, *Parietaria,* Grass mix, *Artemisia vulgaris, Olea europaea, Betula pendula, Cupressus sempervirens* and *Corylus avellana*. These allergens cover the majority of causative agents of respiratory allergy in Italy. In addition we used allergenic extracts of rodents (M and Rt).

Positive (10 mg/ml histamine HCl) and negative (saline solution in glycerine-phenol solution) controls were used as well. SPTs were carried out and interpreted according to international guidelines [20]. The result was read after 15 minutes and expressed as the mean of the major wheal diameter plus its orthogonal. A skin reaction of 3 mm or greater was considered positive.

The profile of the wheals were outlined using a fine-point marking pen and transferred onto patient’s form by adhesive tape.

## Results

**Figure 1:**
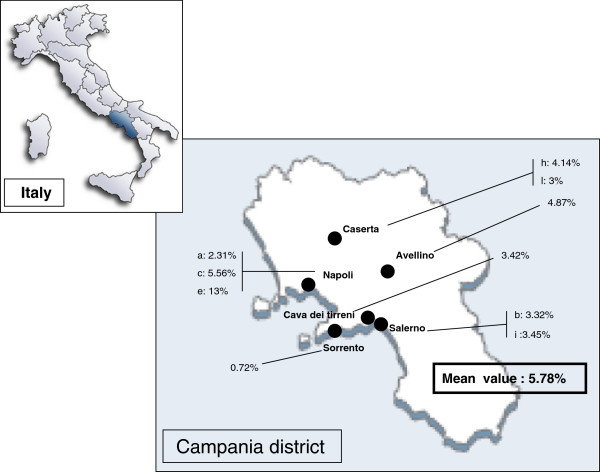
Geographic distribution of the regione Campania centers with the percentages of subjects having positive skin reactions to rodent (mouse/rat) allergens.

**Figure 2:**
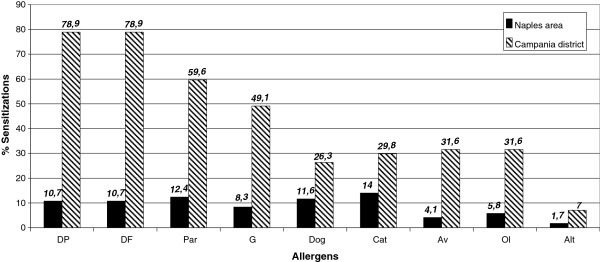
A comparison between associated sensitizations found in Campania district area and in Naples area in a previous study.

A total of 1,477 patients were examined. In this context 985 (66.68%) had a SPTs positivity for at least one allergen and were diagnosed as having respiratory allergy. The 1,477 subjects had a mean age of 31.2 years (range 3–79) and 834 (56.46%) out of them were female. Fifty seven were sensitized to rodents (20 patients only to M, 12 only to Rt and 25 to both M and Rt allergens), 39 patients were females and only 18 males. Thus, the overall sensitization prevalence in subjects with respiratory allergy was 5.78% ranging between 0.72-13% (Figure 1). Only two patients were mono-sensitized to rodents (one to M and one to Rt), both reported only rhinitis. Eleven patients reported rhinitis (R)+bronchial asthma (A), seventeen R+A+conjunctivitis (C), fourteen R+C, nine only A and six individuals only R. Forty three patients exhibited persistent symptoms and fourteen intermittent symptoms. Only fourteen out of 57 patients reported some indoor conditions which constitute predictors for the presence of rodents allergens. In four of these individuals we found the higher levels of cutaneous sensitization to M/Rt, the remaining patients exhibited low/moderate degree of SPT positivity. Since the majority (55/57) of M/Rt sensitized patients showed cutaneous positivity to other common allergens (mites, pollens, moulds and pets) we could not quantify the role of rodents sensitization in eliciting symptoms. The most common sensitizing allergens associated in M/Rt allergic individuals are reported in Figure 2. Dust mite is the first cause of associated sensitization followed by *Parietaria*, Grasses, *Olea europaea* and pet danders. An interesting observation is the high percentage of allergic sensitization to pet (and other animal) dander in individuals with M and Rt allergy found in our previous study in Naples area [11]. This finding is not confirmed in the present survey. The evaluation of M and Rt –serological IgE was not performed because outstanding role of SPT on specific IgE evaluation in discriminating patients sensitized to M allergens [21,22].

The main characteristics of the patients sensitized to M/Rt are summarized in Table 1.

**Table 1:** Characteristics of the 57 subjects with positive SPTs to M and Rt allergens

	**N.**	**%**
SEX (M/F)	18/39	31/68
MEAN AGE	31.2	
AGE RANGE		
- 0-20	19	33.3
- 21-40	25	43.8
- 41-60	8	14.0
➣ 60	5	8.7
+ VE FAMILY HISTORY OF ALLERGY	32 yes/25 no	56.1/43.8
PET AT HOME		
- Cat	3	5.2
- Dog	8	14.0
- None	38	66.6
- Other animals	7	12.2
- Cat + Dog	1	1.7
MODALITY OF EXPOSURE TO M or Rt		
- Positive contact	14	24.5
- Negative contact	43	75.4
SMOKE		
- YES	14	24.5
- NO	43	75.4
CLINICAL SYMPTOMS		
- Rhinitis (R) only	6	10.5
- Asthma (A) only	9	15.7
- Rhinitis + Asthma	11	19.2
- Rhinitis + Conjunctivitis (C)	14	18.6
- R + C + A	17	24.5
SEASONALITY OF SYMPTOMS		
- Intermittent	14	24.5
- Persistent	43	75.4
ASTHMA SEVERITY		
- Mild	15	26.3
- Moderate/severe	42	73.6
MONOSENSITIZED TO RODENTS (M and Rt)	2	3.5
ASSOCIATED SENSITIZATIONS		
- Parietaria	34	59.6
- Dermatophagoides pteronyssinus	45	78.9
- Grasses	28	49.1
- Olive	18	31.5
- Mugwort	18	31.5
- Alternaria	4	7.1
- Cladosporium	1	1.7
- Birch	5	8.7
- Hazelnut	5	8.7
- Dog	15	26.3
- Cat	17	29.8
- Other allergens	5	8.7
DIAMETERS OF RODENTS’- ALLERGEN-INDUCED WHEALS (SPTs)		
	< 6×6 mm (49)	85.9
	> 6×6 mm (8)	14.0

## Discussion

Rodents allergens, especially those of M and to a lesser extent those of Rt, represent a common cause of allergic sensitization and bronchial asthma in children and adult population of US living in inner cities [2,5]. It has been shown that current asthma, defined as having doctor-diagnosed asthma and asthma symptoms in the preceding 12 months, was positively associated to increasing levels of M allergens at home [3]. Recently, Tojusen et al. [23] have shown that every tenfold increase in the bed mouse allergen level was associated with an 87% increase in the odds of any asthma-related health care use among mouse-sensitized, but not among non-mouse-sensitized participants. Furthermore, M sensitization is an independent risk factor for asthma morbidity [24] and M sensitized and exposed children were at higher risk for hospitalization due to asthma [25]. This high rate of allergic sensitization to rodents reflects the high levels of M/Rt allergens in inner-city US houses and schools [5,26-30]. In fact, some environmental conditions such as low-income housing, building-level and neighbourhood- level characteristics are associated to rodents as well as to other pest infestations [31,32].

Because monoclonal antibody-based methods to measure the amount of M/Rt allergens in the dust of indoor environments are not available in Italy, we have no information about the levels of indoor exposure to these allergens. However, Curtis-Brosnan et al. [19] have shown that patient report on the presence of rodents at home and some predictors such as cockroach infestation and poor housing conditions may be sufficient to hypothesize M/Rt allergen exposure in indoor environments.

The results of our study suggest that the prevalence of allergic sensitization to M and Rt allergens is not negligible in urban atopic population living in Campania district area. This rate of sensitization is higher in comparison to that found in Naples area in a previous report [11]. In both studies the main characteristics of M/Rt sensitized individuals (prevalence of female sex, high rate of family history of allergy, periods and type of clinical symptoms) may be easily explained by associated sensitization to other common allergens involved in all individuals. However, no specific symptoms related to exposure to rodents were found in patients with higher degree of cutaneous sensitization to M/Rt and also in two patients mono-sensitized to rodents. The low prevalence of allergic sensitization to M/Rt allergens in our previous study [11] is probably due to the rare reported presence (only in three cases: 13.6%) of environmental conditions commonly considered at high risk for rodent allergens presence [19]. In the present study 14 (24.5%) patients reported ideal conditions for the presence of rodents in indoor environments.

In our previous study in Naples area [11] an important finding was the high prevalence of allergic sensitization to pet (cat/dog) dander in M/Rt sensitized individuals with or without pet contact. This finding confirms our recent report that allergic sensitization to furry animals (cat, dog, horse, guinea pig, rabbit, hamster, cow etc.) may be induced in susceptible individuals with or without animal exposure [33-35]. A possible explanation for high prevalence of mammals sensitization in subjects without known contact with animals could be an indirect exposure or a cross-allergic reaction induced by lipocalins [36] and serum albumin [37] as well as by specific predisposition [38]. In this previous study it is likely that allergic sensitization to rodents in subjects without direct exposure could be induced by these mechanisms. In the present study dust mites, *Parietaria*, grasses, *Olea europaea*, pet danders respectively represent the main associated sensitizing agents (Figure 1), and this finding is in agreement with our previous reports [39,40]. In this case it is likely that a consistent percentage of allergic sensitization to M/Rt could be induced by a true exposure to allergens since environmental conditions are much favourable for the presence of rodents indoors. Moreover, our results suggest that performing a multicenter study at level of district area is more likely to reflect the real rate of allergic sensitization to rodents in Southern Italy in comparison to the rate of the single urban area of Naples.

## Conclusion

In conclusion, the role of allergic sensitization to rodents is not negligible in atopic subjects without occupational exposure living in Campania district area. As a consequence, we suggest that highly atopic individuals and especially those already sensitized to common pet dander be tested by SPTs/evaluation of serum specific IgE to rodents in the case they should begin an occupational exposure to M/Rt or if they wish to keep these animals as pets. Further studies should be carried out in population living in low income areas of our district to explore the possibility that the rate of SPT positivity to rodents be higher in these individuals.

## Competing interests

All authors declare that they have no conflict of interest and that the study has been carried out without any financial support.

## Authors’ contributions

All authors contributed equally to the writing and revision of the manuscript.

## References

[B1] Jeal H Jones M Allergy to rodents: an update Clin Exp Allergy 2010 40 1593 1601 10.1111/j.1365-2222.2010.03609.x 20840394

[B2] Matsui EC Role of mouse allergens in allergic disease Curr Allergy Asthma Rep 2009 9 370 375 10.1007/s11882-009-0054-x 19671380

[B3] Salo PM Jaramillo R Cohn RD London SJ Zeldin DC Exposure to mouse allergen in U.S. homes associated with asthma symptoms Environ Health Perspect 2009 117 387 391 19337513 10.1289/ehp.11847 PMC2661908

[B4] Donohue KM Al-alem U Perzanowsky MS Chew GL Johnson A Divjan A Kelvin EA Hoepner LA Perera FP Miller RL Anti-cockroach and anti-mouse IgE are associated with early wheeze and atopy in an inner-city birth cohort J Allergy Clin Immunol 2008 122 914 920 10.1016/j.jaci.2008.08.034 19000580 PMC2590748

[B5] Matsui EC Simons E Rand C Butz A Buckley TJ Breysse P Eggleston PA Airborne mouse allergen in the homes of inner-city children with asthma J Allergy Clin Immunol 2005 115 358 363 10.1016/j.jaci.2004.11.007 15696095

[B6] Ahluwalia SH Matsui EC The indoor environment and its effects on children asthma Curr Opin Allergy Clin Immunol 2011 11 137 143 10.1097/ACI.0b013e3283445921 21301330

[B7] Wang C Abou El-Nour MH Bennett GW Survey of pest infestation, asthma, and allergy in low-income housing J Community Health 2008 33 31 39 10.1007/s10900-007-9064-6 18080206

[B8] Stelmach I Jerzynska J Stelmach W Majak P Chew G Kuna P The prevalence of mouse allergen in inner-city homes Pediatr Allergy Immunol 2002 13 299 302 10.1034/j.1399-3038.2002.01079.x 12390447

[B9] Onbasi K Ardeniz O Sin AZ Kokuludaq A Sebik F The frequency of mouse and rat allergy among allergic individuals in Izmir (a preliminary report) Allergy 2004 59 1235 1236 10.1111/j.1398-9995.2004.00607.x 15461610

[B10] Forno E Cloutier MM Datta S Paul K Sylvia J Calvert D Thornton-Thompson S Wakefield DB Brehm J Hamilton RG Alvarez M Colòn-Semidey A Acosta-Pèrez E Canino G Celedon JC Mouse allergen, lung function, and atopy in Puerto Rican children PLoS One 2012 7 e40383 Epub 2012 Jul 16 10.1371/journal.pone.0040383 22815744 PMC3398035

[B11] Liccardi G Salzillo A Sofia M Piccolo A Dente B Russo M D’Amato M Stanziola A D’Amato G Sensitization to rodents (mouse/rat) in an urban atopic population without occupational exposure living in Naples, Italy Eur Ann Allergy Clin Immunol 2012 44 200 204 23156068

[B12] Bousquet J and The ARIA Workshop Group Allergic rhinitis and its impact on asthma J Allergy Clin Immunol 2001 108 S147 S334 10.1067/mai.2001.118891 11707753

[B13] Global Initiative for Asthmahttp://ginasthma.com

[B14] Curtin-Brosnan J Paigen B Haqberg KA Langley S O’Neil EA Krevans M Eggleston PA Matsui EC Occupational mouse allergen exposure among non-mouse handlers J Occup Environ Hyg 2010 7 726 734 10.1080/15459624.2010.530906 21058157 PMC3143460

[B15] Krop EJ Doekes G Stone MJ Aalberse RC van der Zee JS Spreading of occupational allergens: laboratory animal allergens on hair-covering caps and in mattress dust of laboratory animal workers Occup Environ Med 2007 64 267 272 17053016 10.1136/oem.2006.028845 PMC2078456

[B16] Bousquet J Michel FB Precision of prick and puncture tests J Allergy Clin Immunol 1992 90 870 872 10.1016/0091-6749(92)90458-E 1460194

[B17] Wever AMJ Wever-Hess J Testing for inhalant allergy in asthma Clin Exp Allergy 1993 23 976 981 10.1111/j.1365-2222.1993.tb00286.x 10779288

[B18] Almqvist C Van Hage-Hamsten M Cat and dog allergens- can intervention studies solve their inscrutable riddle? Clin Exp Allergy 2003 33 1167 1170 10.1046/j.1365-2222.2003.t01-1-01759.x 12956734

[B19] Curtin-Brosnan J Matsui EC Breysse P McCornak MC Hansel NN Tonorezos ES Eggleston P Williams DC Diette GB Parent report of pests and pets and indoor allergen levels in inner-city homes Ann Allergy Asthma Immunol 2008 101 517 523 10.1016/S1081-1206(10)60291-8 19055206 PMC5516632

[B20] Dreborg S Frew A Editors. Position Paper: allergen standardization and skin tests Allergy 1993 48 Suppl 14 49 82 8342740

[B21] Sharma HP Wood RA Bravo AR Matsui EC A comparison of skin prick tests, intradermal skin tests and specific IgE in the diagnosis of mouse allergy J Allergy Clin Immunol 2008 121 933 939 10.1016/j.jaci.2008.01.023 18325579

[B22] Chong LK Ong MJ Curtin-Brosnan J Matsui EC Skin test sensitivity to mouse predicts allergic symptoms to nasal challenge in urban adults Allergy Asthma Proc 2010 31 472 476 10.2500/aap.2010.31.3372 21708058

[B23] Toriusen EN Diette GB Breysse PN Curtin-Brosnan J Aloe C Matsui EC Dose–response relationship between mouse allergen exposure and asthma morbidity among urban children and adolescents Indoor Air 2012 Oct 15 10.111/ina.12009 PMC3562552 23067271

[B24] Moncrief T Kahn R Assa’ad A Mouse sensitization as an independent risk factor for asthma morbidity Ann Allergy Asthma Immunol 2012 108 135 140 10.1016/j.anai.2011.10.003 22374193

[B25] Matsui EC Eggleston PA Buckley TJ Krishanam JA Breysse PN Rand CS Diette GB Household mouse allergen exposure and asthma morbidity in inner-city preschool children Ann Allergy Asthma Immunol 2006 97 514 20 10.1016/S1081-1206(10)60943-X 17069107

[B26] Olmedo O Goldstein IF Acosta L Divian A Rundle SG Chew GL Mellius RB Miller RL Jacobson JS Perzanowski MS Neighborhood differences in exposure and sensitization to cockroach, mouse, dust mite, cat, and dog allergens in New York City J Allergy ClinImmunol 2011 128 284 292 10.1016/j.jaci.2011.02.044 PMC3271316 21536321

[B27] Sheehan WJ Rangsithienchai PA Muilberg ML Rogers CA Chaemghami J Rivard DV Otsu K Hoffman EB Israel E Gold DR Phipatanakul W Mouse allergens in urban elementary schools and homes of children with asthma Ann Allergy Asthma Immunol 2009 102 125 130 10.1016/S1081-1206(10)60242-6 19230463 PMC2658645

[B28] Chew GL Correa JC Perzanowsky MS Mouse and cockroach allergens in the dust and air in northeasther United States inner-city public high schools Indoor Air 2005 15 228 234 10.1111/j.1600-0668.2005.00363.x 15982269

[B29] Permaul P Hoffman E Fu C Sheehan W Baxi S Gaffin J Bailey A King E Chapman M Gold D Phipatanakul W Allergens in urban schools and homes of children with asthma Pediatr Allergy Immunol 2012 23 543 549 10.1111/j.1399-3038.2012.01327.x 22672325 PMC3424376

[B30] Muti D Purohit A Dazy A Verot A De Blay F Mouse (Mus m 1) and rat (Rat n 1) allergen levels in dust from private and public houses in Strasbourg, France are lower than houses in the U.S.A Eur Ann Allergy Clin Immunol 2012 44 93 95 22768731

[B31] Rosenfeld L Chew GL Rudd R Emmons K Acosta L Perzanowsky M Avecedo-Garcia D Are building-level characteristics associated with indoor allergens in the household? J Urban Health 2011 88 14 29 10.1007/s11524-010-9527-4 21274646 PMC3042089

[B32] Rosenfeld L Rudd R Chew GL Emmons K Acevedo-Garcia D Are neighbourhood-level characteristics associated with indoor allergens in the household? J Asthma 2010 47 66 75 10.3109/02770900903362676 20100024 PMC2920139

[B33] Liccardi G Salzillo A Piccolo A Russo M D’Amato G Sensitization to furry animals in an urban atopic population living in Naples, Italy Allergy 2011 66 1500 1501 10.1111/j.1398-9995.2011.02675.x 21790648

[B34] Liccardi G Passalacqua G on behalf of the Allergy Study Group of the Italian Society of Respiratory Medicine (SIMeR): Sensitization to rabbit allergens in Italy- A multicentre study in atopic subjects without occupational exposure Int Arch Allergy Immunol 2006 141 295 299 10.1159/000095435 16940739

[B35] Liccardi G D’Amato G Antonicelli L Berra A Billeri L Canonica GW Casino G Cecchi L Folletti I Gani F Lombardi C Lo Schiavo M Meriggi A Milanese M Passalacqua G Pio R Rolla G Russo M Scaccianoce S Senna GE Scavalli P Scichilone N Sposato B Siracusa A Ventura MT Sensitization to horse allergens in Italy: a multicentre study in urban atopic subjects without occupational exposure Int Arch Allergy Immunol 2011 155 412 417 10.1159/000321414 21346372

[B36] Flower DR North AC Attwood TK Structure and sequence relationships in the lipocalins and related proteins Protein Sci 1993 2 753 761 10.1002/pro.5560020507 7684291 PMC2142497

[B37] Liccardi G Asero R D’Amato M D’Amato G Role of sensitization to mammalian serum albumin in allergic disease Curr Allergy Asthma Rep 2011 11 421 426 10.1007/s11882-011-0214-7 21809117

[B38] Liccardi G Passalacqua G Salzillo A Piccolo A Falagiani P Russo M Canonica GW D’Amato G Is sensitization to furry animals an independent allergic phenotype in non occupationally exposed individuals? J Investig Allergol Clin Immunol 2011 21 137 141 21462804

[B39] Liccardi G Visone A Russo M Saggese M D’Amato M D’Amato G Parietariapollinosis: clinical and epidemiological aspects Allergy Asthma Proc 1996 17 23 29 10.2500/108854196778662381 8814936

[B40] Liccardi G Russo M Piccolo A Lobefalo G Salzillo A D’Amato M D’Amato G The perennial pattern of clinical symptoms in children monosensitized to Olea europaea pollen allergens in comparison with subjects with Parietaria and Gramineae pollinosis Allergy Asthma Proc 1997 18 99 105 10.2500/108854197778605536 9134068

